# Plastination of macroparasites: An eco-friendly method of long-term preservation

**DOI:** 10.14202/vetworld.2017.1394-1400

**Published:** 2017-11-29

**Authors:** Niranjan Kumar, Bhupamani Das, Jayesh B. Solanki, Mehul M. Jadav, Ramasamy Menaka

**Affiliations:** 1 Department of Parasitology, College of Veterinary Science and Animal Husbandry, Navsari Agricultural University, Navsari - 396 450, Gujarat, India; 2 Department of Anatomy, College of Veterinary Science and Animal Husbandry, Navsari Agricultural University, Navsari-396 450, Gujarat, India

**Keywords:** macroparasites, melamine, plastination, preservation

## Abstract

**Aim:**

Preservation of macroparasites by infiltrating the polymer in the tissues can defy the inherited shortcoming of classical wet preservation method.

**Materials and Methods:**

Preservation was done by infiltrating the melamine alone or with xylene (MX)/chloroform (MC)/turpentine oil (MT) in 1:1 and hardener (MH) in 9:1 ratio in the tissues of the gross specimen of the animal parasites.

**Results:**

The plastinated models withstand the process of microbial decomposition, and remain intact in the environmental conditions. The polymer mixture resists the entry of the water molecule, and model dried just after taking out it from the water tank. Overall, the plastinated parasites were dry, non-sticky, glossy, odorless, chemical free, and harmless, to some extent flexible, with detectable morphological structure, and retain their natural form but lost their natural color. Full marks were assigned to the degree of dryness, non-stickiness, and odorlessness to the model plastinated in different solutions on a five-point scale. For flexibility, the score was 1.2, 2.2, and 2.4 for the plastinated model in melamine/MH, MX/MC, and MT solutions, respectively. The average score of glossiness was 4.6 and 5 for the specimen plastinated in melamine/MH and MX/MC/MT solutions, respectively. The degree of dryness, glossiness, stickiness, and flexibility varies non-significantly, with the polymer mixtures used.

**Conclusion:**

The prepared model can be used to educate the students/general mass population.

## Introduction

The macroparasites occupy all the ecological niche of the world and exerts ill effects on the health of the hosts (animals, birds, and human) [1,2]. There are different techniques to preserve the parasites for educational purposes in the academic institutions. The most widely accepted method of preservation is immersions and storage of the biological specimens in 10% formalin or 70% ethyl alcohol [3]. Although these materials are well-known fixative but the stored specimens are having some inherent shortcomings such as wet, with noxious odors, hazardous to the handlers, and difficult to transport. The continuously emitting noxious gas can harm the respiratory system, eyes, and skin of the handlers. Some study highlighted the role of formaldehyde as carcinogenic and neurotoxic agents [4,5]. Histological cross sections are also utilized in some teaching laboratories [6,7]. The disadvantage of histological specimens is their limitation in scope as definitive identification of the parasites requires integration with other factors [8]. The preservatives can also destroy the normal morphological features of the macroparasites, and thus, impede their identification [9-11].

Plastination technique, a method of dry preservation, is a delicate method of forced impregnation by replacing water and lipid tissues with the curable polymers, melamine or others [12-15]. Melamine is a nitrogen-rich heterocyclic triazine used primarily in the synthesis of melamine-formaldehyde resins for the manufacture of laminates, plastics, coatings, commercial filters, glues or adhesives, and molding compounds (dishware and kitchenware) [16,17]. There is a rare report of the inhalation toxicity of the melamine, but its oral exposure may cause the formation of renal calculi [18,19].

The purpose of this study was to develop alternative eco-friendly method of preservation of the macroparasites describing their natural identifying features by infiltrating the melamine polymer.

## Materials and Methods

### Ethical approval

As the study was conducted utilizing the gross specimen of the animal parasites stored in 10% formalin in the departmental museum, so the ethical committee approval was not required.

### Parasites specimens

The whole or segments of parasites belonging to Phylum: Platyhelminthes- Class Trematoda (*Fasciola gigantica*) and Cestoda (*Moniezia* spp.); Nemathelminthes- Class Nematoda (*Ascaris suum*, *Parascaris equorum*, *Toxocara vitulorum*, *Toxocara canis*, *Haemonchus contortus*, *Oesophagostomum* spp., *Bunostomum* spp., *Trichuris ovis*, and *Oxyuris equi*), Arthropoda- Class Insecta (*Oestrus ovis* and blow fly) and Arachnida (soft and hard ticks) have been plastinated.

To measure the extent of shrinkage, the dimension and weight of the plastinating materials were taken before and after impregnation of the polymer.

### Plastinating materials

The melamine polymer, hardener, and touchwood of Asian Paints along with turpentine oil were procured from the local distributors. The acetone, xylene, and chloroform were of Merck make.

### Plastination technique

In the present investigation, plastination technique was performed as per the method described by Menaka *et al*. [20] with certain modification. The selected parasites specimens were washed 24 h in running cold tap water (5°C), to remove as much as possible formalin [21]. For dehydration, the samples were placed in 100% acetone in the ratio 10:1 (approximately) at -20°C. The parasites were dehydrated in 3 changes of dehydrating agents at 1 week interval. When the dehydrating agent’s concentration remained at 99% (approximately after 3 weeks), dehydration was deemed complete [22]. The 3:1 volume ratio of polymer/polymer mixture to the macroparasites specimens was incubated at −20°C for 30 days. To create an ideal plastinated model of the macroparasites, the reagents such as xylene, chloroform, turpentine oil, and hardener were added in different permutation and combination in the melamine. The polymer mixtures were prepared by mixing melamine and xylene (MX), melamine and chloroform (MC), and melamine and turpentine oil (MT) in 1:1 while melamine and hardner (MH) in 9:1 ratio. Following the forced impregnation, the specimens were removed from the polymer/polymer mixture, and kept in a Petri dish for 2-3 days to drain the excess polymer. To impart glossy appearance, the specimens were brushed with colorless varnish.

### Hydrophobicity testing

To check the power of wettability, the plastinated specimen was dipped in water for 30 minutes, and then allowed to stand at the environmental condition.

### Quality evaluation

Five technical personnel were allocated to measure the extent of dryness, non-stickiness, odorlessness, glossiness, and flexibility of the prepared model on a five-point scale.

### Statistical analysis

Results were compiled systematically, and data were analyzed using IBM SPSS Statistics 20.00 for Windows (SPSS Inc., Chicago, USA) to perform Chi-square tests and/or Student’s t-test and/or one-way ANOVA using Duncan test (2-sided) for the determination of statistical significance. The p>0.05 was considered as statistically non-significant.

## Results

The plastinated models withstand the process of microbial decomposition, and remain intact at environmental condition till the acceptance of the manuscript. The polymer mixture resists the entry of water molecules inside the specimens, and the model becomes dry just after taking out it from the water tank; thus, the plastinated model can be maintained even in the environment with high level of humidity.

Overall, the plastinated parasites were dry, non-sticky, glossy, odorless, chemical free, and harmless, to some extent flexible, with detectable morphological structure, and retain their natural form but lost their natural color. On a five-point scale, all five personnel assigned full marks for dryness, non-stickiness, and odorlessness to the model plastinated in different solutions. For flexibility, the score was 1.2, 2.2, and 2.4 for the plastinated model in melamine/MH, MX/MC, and MT solutions, respectively. The average score of glossiness was 4.6 and 5 for the specimen plastinated in melamine/MH and MX/MC/MT solutions, respectively. The degree of dryness, glossiness, stickiness, and flexibility of the prepared model varies non-significantly with the different polymer mixtures.

**Figure-1:**
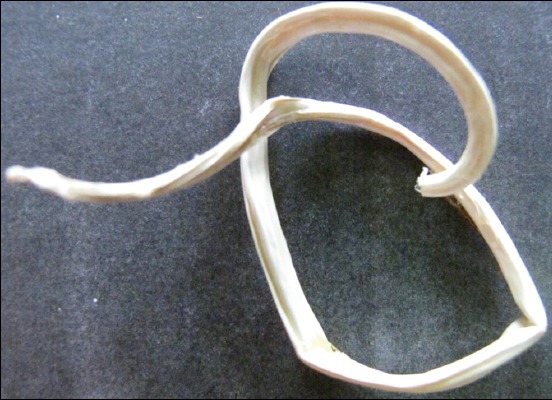
*Parascaris equorum* plastinated in melamine (M) solution.

The parasites plastinated solely in melamine were dry, non-sticky, and odorless but less-glossy and brittle in nature (Figure-1).

**Figure-2:**
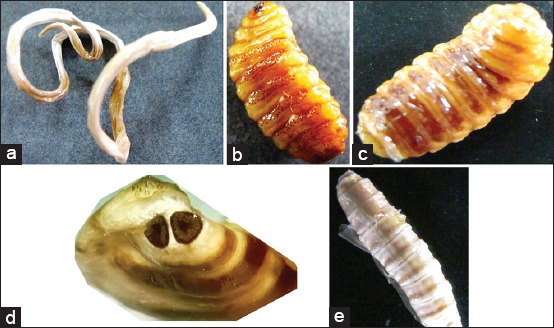
*Ascaris suum* (a); *Oestrus ovis* larva-ventral surface (b); dorsal surface (c), and spiracles (d); blowfly larva-dorsal surface (e) plastinated in melamine and xylene (MX) solution.

The parasites plastinated in MX solution were dry, non-sticky, odorless, and glossy but less flexible (Figure-2a-e). The plastinated insect larvae clearly depicted the feature of immature/mature larvae of *O. ovis*, large, around 2.0 cm long (Table-1) with black oral hooks (Figure-2b), small rows of rose thorn-shaped spines on the ventral aspect (Figure-2b) dark black bands on all the segments on the dorsal aspect (Figure-2c), and truncated and large posterior end with clearly visible brown color “D-” shaped posterior spiracles with radiating slits (Figure-2d). Blow fly larva plastinated in MX solution was elongated (15 mm long×2 mm width), glossy, and creamy color (Table-1 and Figure-2e).

**Table-1:** Shrinkage of macroparasites in terms of dimension and body weight.

Plastination mixture	Parasite										

	Type	Name	Dimension						Weight (mg)		
	
			Length (mm)			Width (mm)			Before	After	Shrinkage (%)
	
			Before	After	Shrinkage (%)	Before	After	Shrinkage (%)			
MX	Round worm	*Ascaris suum*	174	171	1.7	3	2	33.3	270	181.3	32.9
		*Ascaris suum*	161	160	0.6	3	2	33.3	220	143.2	34.9
		*Toxocara canis*	75	71	5.3	1	0.9	10	130	91	30
		Mean±SE	136.66±31.06^a^	134±31.65^a^		2.33±0.67^b^	1.63±0.37^b^		206.67±40.96^c^	138.5±26.17^c^	
	Flat worm	*Moniezia* spp. segments	38	33	13.2	6	5	16.7	270	201	25.5
		*Moniezia* spp. segments	43	40	7.0	6	5	16.7	290	203.7	29.8
		*Fasciola gigantica*	46	40	13.0	7	5	28.6	160	135.6	15.3
		*Fasciolopsis buski*	28	20	28.6	19	15	21.1	330	261.2	20.9
		Mean±SE	38.75±3.94^d^	33.25±4.71^d^		9.5±3.18^e^	7.5±2.5^e^		262.5±36.37^f^	200.38±25.67^f^	
	Insect’s larva	*Oestrusovis* larva	19	16	15.8	7	6	14.3	380	309.8	18.5
		Blow fly larvae	15	14	6.7	2	2	0	50	41.6	16.8
		Mean±SE	17±2^g^	15±1^g^		4.5±2.5^h^	4±2^h^		215±165^i^	175.7±134.1^i^	
	Soft tick	*Ornithodoros* spp.	10	10	0	6	6	0	680	582.7	14.3
MC	Round worm	*Ascaris suum*	190	188	1.1	4	3	25	310	221	28.7
		*Toxocara vitulorum*	165	162	1.8	7	6	14.3	1150	984	14.4
		*Toxascaris leonina*	4	3.5	12.5	0.8	0.6	25	30	10.5	65
		Mean±SE	119.67±58.28^j^	117.83±57.66^j^		3.93±1.79^k^	3.2±1.56^k^		496.67±336.52^l^	405.17±295.73^l^	
	Flat worm	*Moniezia* spp. segments	32	30	6.25	6	5	16.7	260	181.6	30.2
		*Fasciola gigantica*	32	28	12.5	6	3	50	150	109.6	26.9
		Mean±SE	32±0^m^	29±1^m^		6±0^n^	4±1^n^		205±55o	145.6±36^o^	
	Insect’s larva	*Oestrus ovis* larva	16	14	12.5	6	4	33.3	220	151	31.4
		*Oestrus ovis* larva	17	11	35.3	7	5	28.6	180	145.9	18.9
		Blow fly larvae	18	17	5.6	2	1.6	20	52	47.1	9.4
		Mean±SE	17±0.58^p^	14±1.73^p^		5±1.53^q^	3.53±1.01^q^		150.67±50.67^r^	114.67±33.82^r^	
	Insect	*Tabanus* spp.	18	18	0	4	4	0	60	21	65
	Hard tick	*Ixodes* spp.	9	9	0	4	4	0	90	90.8	0.9
MT	Round worm	*Ascaris suum*	134	131	2.2	2	1	50	160	101.4	36.6
		*Toxocara canis*	4.5	4	11.1	0.9	0.7	22.2	32	12.5	60.9
		Mean±SE	69.25±64.75^s^	67.5±63.5^s^		1.45±0.55^t^	0.85±0.15^t^		96±64^u^	56.95±44.45^u^	
	Flat worm	*Moniezia* spp. segments	30	27	10	6	5	16.7	220	195.5	11.1
		*Fasciola gigantica*	47	41	12.8	6	3	50	160	126.4	21
		Mean±SE	38.5±8.5^v^	34±7^v^		6±0^w^	4±1^w^		190±30^x^	160.95±34.55^x^	
	Insect’s larva	*Oestrusovis* larva	18	13	27.8	7	5	28.6	190	156.7	17.5
			18	15	16.7	7	6	14.3	360	283.5	21.3
		Mean±SE	18±0^y^	14±1^y^		7±0^z^	5.5±0.5^z^		275±85^aa^	220.1±63.4^aa^	
	Hard tick	*Ixodes* spp.	10	10	0	6	6	0	130	131.2	0.9
MH	Insect’s larva	*Oestrus ovis* larva	21	17	19.0	8	5	37.5	200	176	12
	Hard tick	*Ixodes* spp.	10	10	0	6	6	0	190	192	1.1

SE=Standard error; Significant at p≤0.05; p value a=0.95, b=0.41, c=0.23, d=0.41, e=0.64, f=0.21, g=0.47, h=0.89, i=0.87, j=0.98, k=0.77, l=0.85, m=0.10, n=0.18, o=0.46, p=0.18, q=0.47, r=0.59, s=0.99, t=0.40, u=0.67, v=0.72, w=0.18, x=0.59, y=0.06, z=0.10, aa=0.66; MX=Melamine and xylene, MC=Melamine and chloroform, MT=Melamine and turpentine oil, MH=Melamine and hardener

**Figure-3:**
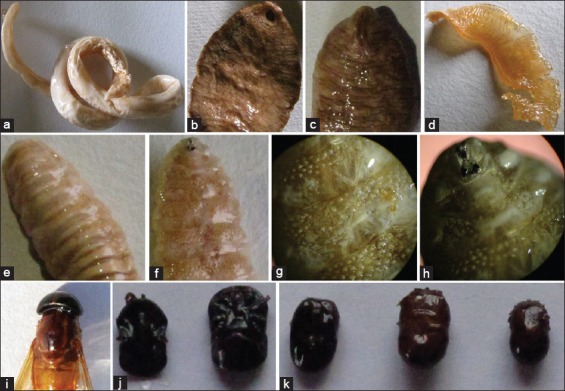
*Toxocara vitulorum* (a); *Fasciolopsis buski*-ventral surface (b); and dorsal surface (c); *Moniezia expansa* (d); immature *Oestrus ovis* larva-ventral surface (e); dorsal surface (f); ventral surface close view (g); and anterior end close view (h); *Tabanus* spp. fly (i); *Ornithodoros moubata* soft tick (j); *Rhipicephalus* (*Boophilus*) *microplus* hard tick (k) plastinated in melamine and chloroform (MC) solution.

The parasites plastinated in MC solution were dry, non-sticky, odorless, and glossy while to some extent flexible (Figure-3a-k). The plastinated *T. vitulorum* was up to 16.5 cm×7 mm with translucent cuticle, and the body does not taper toward the extremities (Table-1 and Figure-3a). The large thick-set fluke plastinated in the MC solution described the overall morphological features of *Fasciolopsis buski* like elongate-oval body slightly broader posteriorly than anteriorly without shoulders, measuring about 30 mm by 20 mm, much larger ventral sucker than the oral one (Figure-3b and c). *Moniezia* spp. segments plastinated in MC solution were transparent, about 6 mm in width, broader than long, with two sets of genital organs with the marginal genital pores (Table 1 and Figure-3d). The plastinated immature larvae of *O. ovis* was around 1.6 cm long (Table-1), with truncated and large posterior end (Figure-3e), small rows of rose thorn-shaped spines on the ventral aspect (Figure-3f and g), and black oral hooks (Figure-3h). *Tabanus* spp. fly plastinated in MC solution depicted the important morphological structure, large compound eyes, longitudinal white stripes on the thorax, clear wings with the 4^th^ radial vein being forked at the apex, hexagonal discal cell, and brown color abdomen (Figure-3i). The plastinated soft (*Ornithodoros moubata*) and hard [*Rhipicephalus* (*Boophilus*) *microplus*] tick in MC solution had described their common morphological features (Figure-3j and k).

**Figure-4:**
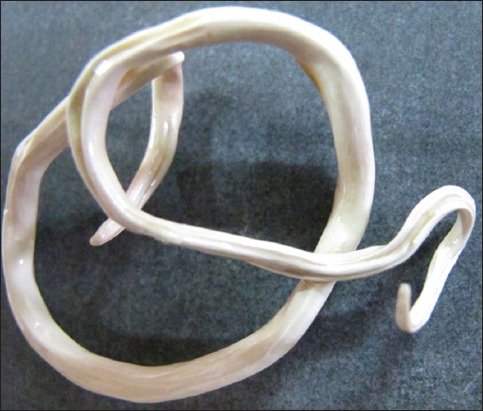
*Ascaris suum* plastinated in melamine and turpentine oil (MT) solution.

**Figure-5:**
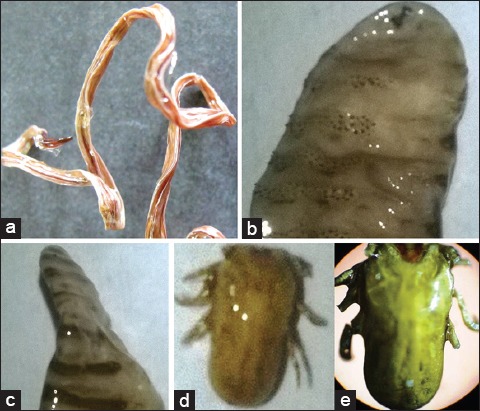
*Ascaris suum* (a); immature *Oestrus ovis* larva-ventral surface (b) and lateral surface (c); *Rhipicephalus* (*Boophilus*) *microplus* (d and e) plastinated in melamine and hardener (MH) solution.

The parasites plastinated in MT solution were dry, non-sticky, odorless, glossy, and flexible (Figure-4). The parasites plastinated in MH solution were hard, brittle, and difficult to handle during the plastination process (Figure-5a-e). The set protocol and plastinating agent failed to yield the plastinated model of small size nematodes such as *H. contortus*, *Oesophagostomum* spp., *Bunostomum* spp., *T. ovis*, and *O. equi*.

Shrinkage of macroparasites in terms of dimension and body weight is summarized in Table-1. The parasites with soft body had received more shrinkage than the parasites with tough body. The tough chitinous layer (exoskeleton) of the arthropods withstands the process of shrinkage in various plastinating polymer solutions except MH. The specimens plastinated in MH polymer solution had received drastic overall shrinkage, and become brittle in nature (Figure-5a). Shrinkage of the round/flat worm and insect larva in terms of length, width, and weight was statistically non-significant in MX, MC, and MT plastination mixture (Table-1).

## Discussion

Plastination technique was originally developed for the preservation of biological specimens in the medical world by Gunther von Hagens in 1977 [23]. The macroparasite, *Ascaris lumbricoides*, was first time plastinated by Asadi and Mahmodzaeh [22] through Sl0 Techniques. The alcohol or formaldehyde persevered parasite samples have many disadvantages such as being less permanent, having regular needs of changing the immersion solution, the unpleasant smell, and having hardly recognizable parts of the parasites [14]. Plastinated parasites can be an excellent alternative as it lowers the risk of undue exposure to the formaldehyde with higher health and safety regulations. Furthermore, the plastinated model is easy to carry, palpable, with clearly visible structure, and can be stored for an infinite period at room temperature [12].

The present study dealt with the preparation of a plastinated model of macroparasites of animal origin using melamine polymer first time in India. There is a very limited report of using melamine polymer as plastinating agents to preserve the biological specimen in native condition [20].

Laboratory grade xylene is a fair mixture of o, p, m, and p isomer and ethylbenzene (6-20%) with the traces of toluene, trimethylbenzene, phenol, thiophene, pyridine, and hydrogen sulfide. The xylene has multivalent function, as a plasticizing agent (p-isomer is the precursor to terephthalic acid and dimethyl terephthalate, used in the production of polyethylene terephthalate plastic bottles and polyester clothing), mixture of o and p isomer used as a solvent (in the printing, rubber, and leather industries) for thinning paints and varnishes, as a cleaning/clearing agent (for steel, silicon wafers, integrated circuits, ear wax, and dentistry, the removal of immersion oil from objective lens/slide, and the removal of paraffin wax in the histopathology), as a cooling agent, and as an inflammable substance. Kandyala *et al*. [24] concluded that the high solvency of xylene/chloroform renders the tissue transparent, and enhancing the infiltration of the polymer, this feature was also noted in the specimen plastinated in the MX solution in the present investigation.

The experiment was performed under low temperature of −20°C as it fixes the shape of specimens with minimum shrinkage, and further prevents its decomposition. Forced impregnation of the polymer inside the biological specimen is generally performed under vacuum/low pressure resulting in the severe shrinkage of the specimen, so the present experiment was conducted on environmental pressure to avoid this undue shrinkage, and a non-significant level of shrinkage of the round/flat worm, and insect larva in terms of length, width, and weight was noted in MX, MC, and MT plastination mixture. Sagoo and Adds [25] observed the phenomenon of shrinkage in the plastinated brain slice using Biodur TM S10/S3 polymer, and concluded 6.99% and 6.19% shrinkage in lengths and widths, respectively, was acceptable.

The plastinated specimens in the different polymer mixtures were dry and odorless, and successfully demonstrated the gross morphological features of the parasites while the color was more esthetic than that of the specimens stored in formaldehyde. The shrinkage and resulting distorted anatomy were the important observations of the present investigation, and were also observed by Miklošova and Mikloš [26] in the most ideal plastination method, silicone S10. Furthermore, Latorre *et al*. [27] also concluded shrinkage and color changes as the major causes of failures of the plastination technique.

The water molecule is the key player to start the process of microbial decomposition. The polymer mixtures of the present investigation critically resisted the entry of water molecules inside the specimens, thus exhibiting the antimicrobial features [28].

## Conclusion

The plastinated parasitological model can be an excellent educational material for teaching the macroparasites, and technique can be used an alternative method of traditional preservation. Parasites with soft body cover require further standardization of the plastinating technique. To enhance the flexibility in the plastinated specimen, some suitable plasticizing agents should be tried in the technique.

## Authors’ Contributions

NK planned and accomplished the overall research work. BD and MMJ extended their physical support in performing the plastination technique. NK did the data analysis, drafted, and revised the manuscript. JBS did the initial revision of the manuscript. RM had extended the technical help in doing the plastination technique. All authors read and approved the final manuscript.
